# Investigating the Impact of Cell Inclination on Phase Change Material Melting in Square Cells: A Numerical Study

**DOI:** 10.3390/ma17030633

**Published:** 2024-01-28

**Authors:** Farhan Lafta Rashid, Abbas Fadhil Khalaf, Mudhar A. Al-Obaidi, Anmar Dulaimi, Arman Ameen

**Affiliations:** 1 Petroleum Engineering Department, College of Engineering, University of Kerbala, Karbala 56001, Iraq; farhan.lefta@uokerbala.edu.iq (F.L.R.); abbas.fadhil@uokerbala.edu.iq (A.F.K.); 2 Technical Institute of Baquba, Middle Technical University, Baquba 32001, Iraq; dr.mudhar.alaubedy@mtu.edu.iq; 3 Technical Instructor Training Institute, Middle Technical University, Baghdad 10074, Iraq; 4 Department of Civil Engineering, College of Engineering, University of Kerbala, Karbala 56001, Iraq; 5 College of Engineering, University of Warith Al-Anbiyaa, Karbala 56001, Iraq; 6 Department of Building Engineering, Energy Systems and Sustainability Science, University of Gävle, 801 76 Gävle, Sweden

**Keywords:** Phase Change Materials (PCMs), angle of inclination, melting process, melting completion time, paraffin wax

## Abstract

In order to determine the ideal degree of inclination that should be employed for constructing effective thermal energy storage systems, it is important to examine the impact of inclination angle on the melting behavior of phase change materials (PCMs) such as paraffin wax within a square cell. In consequence, this would guarantee the greatest capacity for energy release and storage. Additionally, analyzing this influence aids engineers in creating systems that enhance heat flow from external sources to the PCM and vice versa. To find out how the cell’s inclination angle affects the melting of PCM of paraffin wax (RT42) inside a square cell, a numerical analysis is carried out using the ANSYS/FLUENT 16 software. Specifically, the temperature and velocity distributions, together with the evolution of the melting process, will be shown for various inclination angles, and a thorough comparison will be made to assess the influence of inclination angle on the PCM melting process and its completion. The findings demonstrated that when the cell’s inclination angle increased from 0° to 15° and from 0° to 30° and 45°, respectively, the amount of time required to finish the melting process increased by 15%, 42%, and 71%, respectively. Additionally, after 210 min of operation, the PCM’s maximum temperature is 351.5 K with a 0° angle of inclination (horizontal) against 332.5 K with an angle of inclination of 45°.

## 1. Introduction

Phase change materials (PCMs) are utilized in an extensive variety of applications, from semiconductors to structures, to store energy and minimize temperature changes. PCMs have the ability to absorb, store, and release huge amounts of thermal energy. The utilization of PCMs in thermal energy storage systems (TES) is recognized as an effective method for conserving available energy. PCMs are a research subject that is relevant to many academics due to their high energy storage density. Plenty of investigations have been conducted up to this point to look at the use of PCMs in various fields. Among the uses for PCMs are solar energy storage [1,2,3], battery thermal management systems [4,5,6], electronic cooling [7,8], and building energy conservation [9,10].

The processes of heat and mass transport determine the characteristics of materials (microstructure, strength, etc.) when phase transition occurs. The reason PCMs have drawn interest is due to their capacity to store and release latent heat, which can be utilized for thermal management and saving energy. There are numerous uses for the energy that PCMs may emit or store. The degree of achievement fluctuates with PCM, but three critical characteristics have been identified: (i) thermal conductivity, (ii) latent heat, and (iii) phase transition temperature. Energy storage components lessen the imbalance between demands and supplies, thereby increasing system efficiency with regard to energy [11]. Several procedures were used to enhance the heat transfer of PCMs in porous media. The following illustrates some successful samples from the open literature with details.

In metal foam composite PCMs, Tian and Zhao (2011) [12] found a solution to the combined heat transfer issue caused by natural convection and heat conduction in the liquid and phase change zones. The overall heat transfer efficacy of PCM was enhanced even though metal foam inhibited natural convection. It was found that the improvement in thermal conduction compensation was greater than the loss from natural convection.

An experimental investigation was conducted by Korti and Guellil (2020) [13] to elaborate on the impact of tilt on the thermal performance of PCM melting within a square cavity. Consideration was given to the inclination angle range of 0° (vertical), 45° (inclined), and 90° (bottom). As PCM, paraffin with a melting point between 49 and 54 °C was utilized. A sizable void developed in the free surface PCM air as a result of the volumetric shrinkage of PCM throughout the solidification phase prior to the melting tests. Using an infrared camera and thermocouples to measure temperatures, the impact of inclination and the shrinkage event were quantitatively examined. The melting front development, the rate of heat transfer, and the inclination angle are all significantly impacted by the natural convection behavior, according to the results. The average total melting times for the examples with inclined and bottom cavities were 48% and 56% shorter than those with vertical cavities, respectively.

In order to investigate the implications of various tilt angles on the melting property of PCM used as a passive cooling material inside a rectangular container, Abdulmunem et al. (2020) [14] published empirical and mathematical analyses. The four angles at which the experimental and numerical testing are conducted are 0°, 30°, 60°, and 90°. One of the walls of the rectangular container containing PCM is heated to a steady temperature of 373 K. The acceptable error ratios, which ranged from 4 to 6%, were in good match with the empirical and mathematical findings. The findings further clarified that a decline in the tilt angle of the PCM container can end up in an extension of the time required for the PCM to melt.

Groulx et al. (2020) [15] achieved a quantitative examination of the melting of a PCM within a rectangular enclosure that may have fins and an inclined surface. The goal of this study is to control the temperature of a PCM-filled, finned PV panel that has been fitted at various tilt angles. The system under study was represented as a 2D rectangular enclosure stuffed with PCM (RT25) and positioned between two aluminum plates. The enclosure’s front was subjected to a continuous heat flux of 1000 W/m^2^ for a duration of two hours. Four distinct designs were taken into consideration: a PCM enclosure without fins, a PCM enclosure with one cantered full-width fin, one half-width fin fastened to the front plate, and one half-width fin connected to the rear plate. The findings demonstrated that when a full-width fin is installed in the PCM enclosure and is concurrently connected to the front and rear plates, the PV-PCM panel’s thermal management is at its most effective. With this type of PV panel design, early on natural convection heat transmission from both sides of the PCM enclosure controls the PCM melting process, with additional heat losses from the back plate to the surrounding air.

Using fin inclinations, Fekadu and Assaye (2021) [16] increased the melting rate of PCMs on three adiabatic and one isothermal side of a rectangular enclosure. To enhance the PCM melting rate efficiency within a rectangular enclosure, the number of fins was improved while maintaining the enclosure’s same length and width and maintained fin thickness of 4 mm. As the number of fins in the enclosure rose, the length of the fins was lowered to maintain the exact amount of PCM inside. Additionally, a consistent temperature of 333.5 K was retained from the bottom side of the walled enclosure. These numerical studies were conducted with aluminum fins. It was discovered that a case of two fins was the ideal number in this situation. Analyses on the ideal number of fins for this scenario’s melting rate increase were then conducted using three different fin inclination angle scenarios (60°, 45°, and 35°). Specifically, the optimum fin number of two fins can minimize the melting time by 43% when contrasted with pure melting PCM.

Li et al. (2021) [17] performed a numerical analysis of the graded metal foam composite phase change materials (CPCMs) heat storage capacities. The body-cantered-cubic unit was created as a representation of the intricately linked structure of metal foam. The authors constructed seven models with varying inclinations between 0° to 180° in order to better explain the impact of heat flux orientation. Concurrently, three distinct forms of metal foams—uniform, negative, and positive porosity—were constructed to improve the melting process and examine its impacts. The melting actions of similar models with varying inclinations were compared, and it was discovered that the melting behaviors are significantly influenced by the heat flux directions or model inclinations. In comparison to models with small inclinations (μ < 90°), those with greater inclinations (μ > 90°) have quicker melting rates. Furthermore, the conclusion that the negative model is quite beneficial to the charging process was reached by contrasting the models with various graded structures. The negative models have decreased in the entire melting times by 11.06% when contrasted with those uniform models when the models’ inclinations are 120° and 150°.

The melting behavior of a paraffin/copper foam composite (PCM heat storage unit) with a rectangular encapsulation under the influence of various inclination angles was investigated by Huang et al. (2022) [18]. The dynamic computational models with linked heat conduction and natural convection were resolved mathematically, and the outcomes were confirmed with experimental data collected from published studies. The findings showed that while spontaneous convection is strongly suppressed by the ligaments of metal foams, the thermal characteristics and melting capabilities of composite PCM systems vary depending on the tilt angle. For tilted container systems, particularly the vertical case, natural convection appeared to be more powerful and was entirely eliminated in the horizontal cases (θ = 180° and 0°). A dead zone for heat transfer developed at the bottom of the tilted container as a consequence of natural convection. In comparison to the other tilting examples, the horizontal cases had the largest boundary heat fluxes during the later stages of melting and a shorter total melting time.

Variji et al. (2022) [19] performed computational modeling to investigate the impact of inserting metal foam on heat transfer via PCM and the electrical efficiency of PV-PCM systems. The impacts of metal foam with porosities ranging between 0.7–0.9 were compared. In addition, a range of inclination degrees, from 0° to 90°, were taken into account in order to assess the impact of inclination angle on the augmentation of natural convection via liquid phase change material. The findings showed that improved heat transmission through PCM and improved natural convection of molten PCM would come from raising the inclination angle. Moreover, the PV-PCM system increases average temperature and electrical performance by 6.8% and 9.8%, respectively, when metal foam with a porosity of 0.9 is added.

Using ANSYS FLUENT 17.2 and the finite volume approach with the enthalpy-porosity methodology, Alnakeeb et al. (2023) [20] created a 3D numerical model assuming the identical mass of PCM and the area of heat transfer while varying the aspect ratio. Positive comparisons were found between the numerical model’s estimates and the related data from experiments. Through numerical analysis, the temperature distribution, velocity vectors, melt fraction, and total melting times were examined. For aspect ratios of 0.4, 0.6, 0.8, and 1, the corresponding minimal total melting times happened at inclination angles of 30°, 45°, 75°, and 90°. Additionally, the optimal state has an inclination angle of 30° and an aspect ratio of 0.4.

In their study, Khademi et al. (2023) [21] compared the new combination system that uses water as an auxiliary fluid with inclined systems, which are amongst the less expensive techniques. At the conclusion of the process of melting, oleic acid was nominated as an insoluble PCM in water so that it could be used in additional TES cycles. By applying water directly over PCM, the combined system made use of the variations in densities between the two materials to accelerate the melting process of PCM. The energy storage rate was first determined by analyzing the inclined system at seven distinct inclination angles. This water-PCM system’s continuity, momentum, and energy formulas were used to create a mathematical model. Following that, an analysis of the combination system’s melting process was conducted to assess the rate of energy storage between inclined and combined systems. There is a surge in convection when contrasting the average fluid velocity of the combined system with the optimum case of inclined systems, which is 5.44 times greater. It was also discovered that the inclined system’s energy storage rate (0.162 kW/kg at a 30° inclination) is 1.85 times lower than that of the combined system (0.299 kW/kg).

A critical analysis of the above-demonstrated studies would ascertain the significance of the recent study. Specifically, the impact of the inclination angle on melting the PCMs within a square cell has not been yet investigated. To develop systems that use PCMs for various applications such as energy storage or temperature regulation, it is important to understand the consequence of the inclination angle of the cell containing PCM. Furthermore, it is essential to investigate how long the melting process takes under various inclination degrees as it would shed light on the length of time needed for PCM-based systems to become operational. Accordingly, this study comes to fill this gap in the open literature. In other words, the impact of the inclination angle on the melting of PCMs within a square cell. To systematically conduct this study, a specific set of continuity, momentum, and energy equations will be considered to represent the two dimensions modeling of the melting process of PCMs. Accordingly, the evolution of the melting process and temperature and velocity distributions will be represented for different inclination angles with a comprehensive comparison to evaluate the impact of inclination angle on the melting process of PCM and the completion of the melting process. It can be said that the associated results of this study would aid engineers in developing systems that are more effective and efficient by understanding how the melting process is affected by the angle of inclination.

## 2. Numerical Procedure

### 2.1. Physics Models

**Figure 1:**
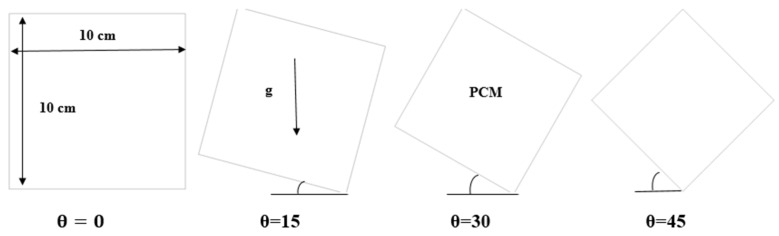
Representation of physical model.

The cell that was studied is a square cell with a side length (10 cm) while considering several angles of (0°, 15°, 30°, and 45°). The heat source is from all directions, as shown in Figure 1. In this regard, it should be noted that the inclination angle of 90° has been excluded. When the inclination angle in a square cell is 90°, one side of the square will be in direct alignment with the direction of gravity. Because gravity does not provide natural convection currents or appreciable fluctuations in the flow of the melting material in such a situation, investigating the phase change material melting process is rendered less effective. Thus, it is practical to exclude an inclination angle of 90° to guarantee a more significant and representative investigation. The range of inclination angles between 0° and 45°, includes those where the phase change process is significantly impacted by gravitational factors.

### 2.2. Computational Procedure

The numerical analysis gives the ability to predict details of the melting processes that occur in the half-cylindrical cell. The flow is determined to be two-dimensional, laminar, incompressible, and unstable. To systematically represent the melting process, the liquid and solid phases are assumed to be isotropic, homogenous, and within a thermal equilibrium at the interface. Enthalpy-porosity path was adopted for the PCM’s phase-change region. The solid-liquid interface’s continuous mobility, temporal conduct, and nonlinearity make the melting processes of PCMs unique to congregations. The melting processes of PCMs are modeled by allowing for the simultaneous continuity, momentum, and energy governing partial differential equation as represented in Equations (1)–(3), respectively [22,23,24,25]:

(1) $$\frac{\partial \rho }{\partial t}+\nabla \;.\left(\rho V\right)=0$$

(2) $$\frac{\partial \left(\rho v\right)}{\partial t}+\nabla \;.\left(\rho V\right)=-\nabla P+\mu \nabla^{2}V+\rho g+S$$

(3) $$\frac{\partial }{\partial t}\left(\rho H\right)+\nabla .\left(\rho VH\right)=\nabla .\left(K\nabla T\right)$$

ρ is density, t is time, V is velocity, μ is dynamic viscosity, S is the source term, H is specific enthalpy.

The total of the sensible enthalpy (*h*) and the latent heat (Δ*H*) is the specific enthalpy (*H*).

(4) H=h+ΔH

(5) $$h=h_{ref}+\int_{T_{ref}}^{T}C_{p}dT$$

(6) $$\nabla H=\beta L_{f}$$

The liquid fraction (*β*) can be conveyed as follows, with the latent heat ability ranging from zero for a solid to one for a liquid [26,27,28]:

(7) $$\beta =\left\{\begin{array}{c} 0\;solidus\;if\;T<T_{s}\\ 1\;liquidus\;if\;T>T_{l}\\ \frac{T-T_{s}}{T_{l}-T_{s}}if\;T_{s}\leq T\leq T_{l} \end{array}\right\}$$

Due to the phase change influence on the convection, the momentum equation gains a source term (*S*) that is Darcy’s law damping term. The counter of Equation (8) clarifies the source term in the equation for momentum.

(8) $$S=\frac{{C(1-\beta )}^{2}}{\beta^{3}}V$$

The shape of the melting forepart is reflected by the mushy zone constant (*C*). *C* varies between 10^4^ to 10^7^. In the current study, *C* is set to be 10^5^.

### 2.3. Boundary Conditions

The studied square cell is insulated on three sides, and the remaining side is the one through which the heat passes to the PCMs. The temperature (80° C) is passed into the wall. A paraffin wax (RT42) is used as a PCM. The thermo-physical properties of paraffin are listed in Table 1.

### 2.4. Assumptions

The following assumptions are considered in the current study to evaluate the melting processes inside a rectangular cell:

- A 2-D model is used to represent the mathematical formula of the melting processes,
- Fixed thermal characteristics of the PCM in both the solid and liquid phases,
- The flow is unsteady, laminar, and incompressible,
- The viscous dissipation term is unimportant.

The outcomes of the solid-liquid phase transition’s volume change are disregarded, and there is no heat gain or loss from the environment.

**Figure 2:**
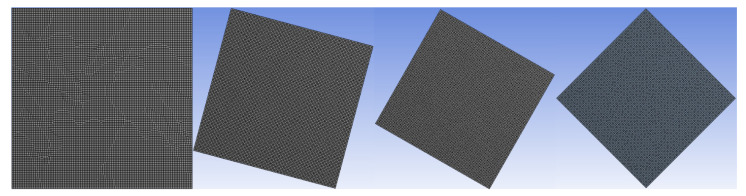
Configuration of the mesh model.

Figure 2 shows the mesh distribution for the model being studied.

### 2.5. Grid Independent Test

**Figure 3:**
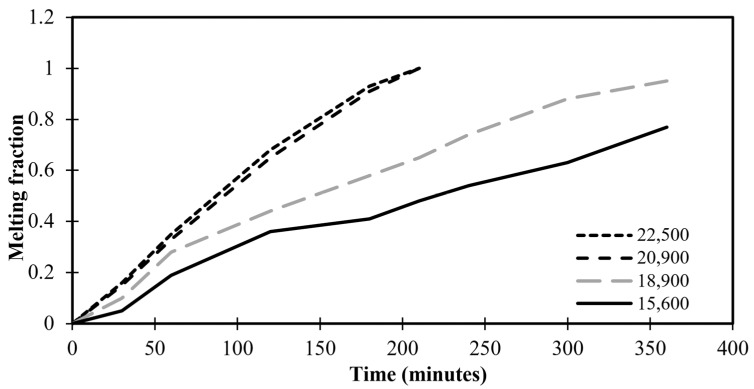
Melting fractions against time for different grid numbers.

Grid convergence is applied to define the development of the results by utilizing successively smaller sizes of grids for the computations. As the mesh gets finer, a calculation should get closer to the true answer. The standard grid-independence analysis CFD technique begins with a coarse mesh and progressively fine-tunes it until the changes seen in the results are less than a predetermined allowable error. To make sure the answer is independent of the chosen grid size, many meshing sizes—15,600, 18,900, 20,900, and 22,500 grids—are investigated for this specific reason. The time-variation of volumetric melt fraction for various numbers of grids is illustrated in Figure 3. Figure 3 depicts the variation of the melting time as insignificant above the 22,500 grids of the meshing system. Therefore, this value of grids is considered throughout the present numerical study.

## 3. Validation

**Figure 4:**
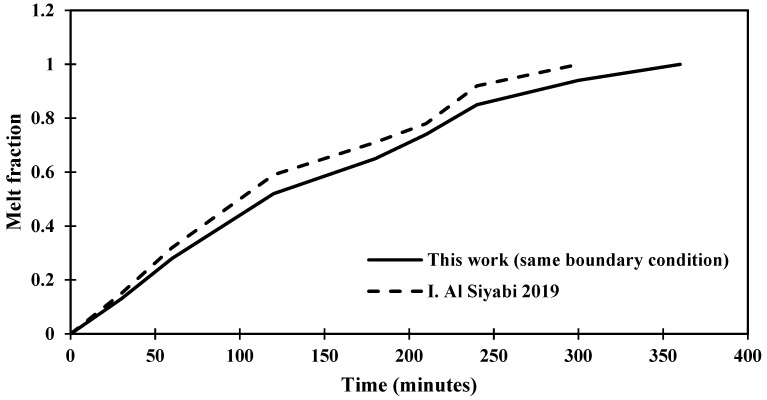
Distinction of the melting fraction versus operating time for an inclination angle of 45° for this study against the research [29].

The validation of the solution is verified through comparison with a previous study, as presented in Figure 4, which shows the melting process that affected by the degree of inclination of the shape to be studied and that the change in the time of the melting process is a result to the variance in the area of the shape and the temperature.

## 4. Results and Discussion

In this study, four cases were studied to clarify the consequence of the angle of inclination of the cell on the melting process besides evaluating the time essential to complete the melting process.

### 4.1. Case One (Angle = 0°)

**Figure 5:**
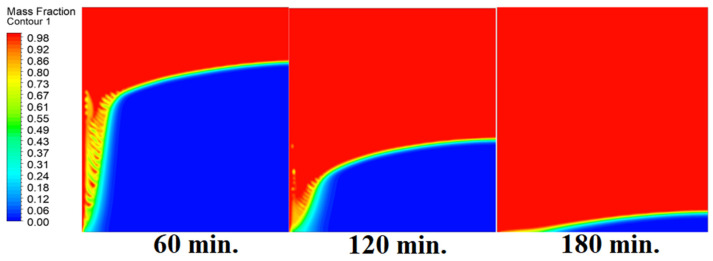
Progression of the melting process predicted with 0°.

In this case, the cell is studied at a zero inclination angle. Figure 5 shows that the melting process commences with the impact of the conductive load that is along the wall. Specifically, the melting process begins with the effect of conduction from the heat source here, which is the wall. The heat transfer then begins with natural convection. Thus, the direction of the melting process tends to start from the left side of the 2D cell as it is the source of heat, besides having the effect of gravity, and the last is the density difference of the substance after melting. In other words, as the phase-changed materials move away from a wall, it becomes clear that the melting process also is dependent on the natural load. More specifically, because heat transfer depends on natural convection, the melting process slows down as we go further away from the wall.

**Figure 6:**
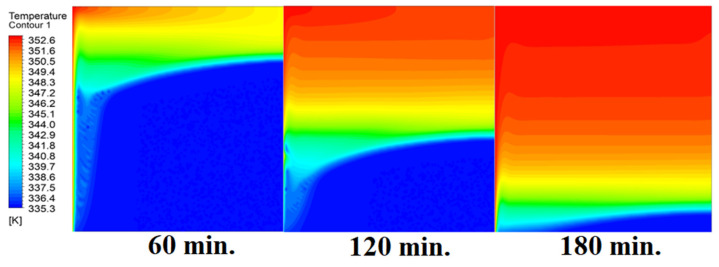
Temperature distributions against operational time with 0°.

The analysis of the interaction between conduction and natural convection processes provides a full explanation for the observed patterns in the temperature distribution inside the PCM within the square cell at zero inclination angles. Figure 6 illustrates different patterns of temperature distribution inside PCM at different operational times at zero inclination angles. Conduction is the primary heat transfer mechanism during the early phases of the melting process when the cell is at a zero inclination angle. Because of the direct contact with the heat source, the temperature distribution is faster close to the heated wall. The temperature in the immediate area of the wall rises quickly as a result of heat transfer via the PCM. This is comparable to the “conduction load”, in which direct interaction between molecules effectively transmits heat energy. In other words, the temperature distribution is faster as it depends firstly on the conduction load; however, it slows down as we move away from the wall because it depends on the natural load. With increasing time, the conduction load decreases, and the effect of natural convection increases. Temperature differentials and PCM density gradients are what cause natural convection. As the material near the wall becomes warmer, it becomes less dense and rises, creating a convective flow. In contrast, the ascending warmer material is replaced by the lower-lying, colder PCM from the interior. The temperature distribution moves slowly away from the wall as a result of this mechanism. A complex pattern of temperature distribution is produced when a conduction-dominated regime gives way to one dominated by natural convection. The temperature rises more slowly at first near the wall, then more quickly as natural convection takes control. At zero inclination angle, the observed changes in temperature distribution over time are a result of the interaction between these heat transmission mechanisms. This physical interpretation explains the dynamic behavior of PCM melting in accordance with the basic laws of heat transport.

**Figure 7:**
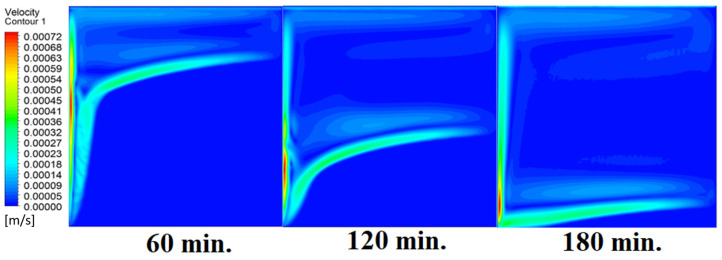
Velocity distributions against operational time with 0°.

Our comprehension of the dynamic behavior is further improved by the velocity distribution versus operational time that can be seen during the PCM melting process inside a square cell. In the early stages of operation, the velocity distribution tends to be greater in the vicinity of the heated wall. In other words, the maximum speed can be noticed near the wall. This phenomenon is directly related to the dominating conduction mechanism’s quick temperature rise. A convective flow is started as the PCM next to the wall changes phases and melts into a liquid. The molten PCM, also known as magma, moves in a more noticeable manner as natural convection takes control as operating time increases. The velocity distribution is seen to change from a concentrated pattern close to the wall to a more scattered pattern, which indicates the change from a conduction-dominated regime to a naturally occurring convection-dominated regime. The molten PCM moving away from the wall is a sign of the convective currents in the PCM changing over time. The shift in the velocity distribution is important because it signals the start of the material change, which is the point at which the molten PCM starts to move around and affects the system’s overall temperature distribution. Figure 7 presents the velocity distribution against operational time, which initially assures the movement of magma (molten PCM), which is initially close to the wall and then moves to the stage of material change.

### 4.2. Case Two (Angle = 15°)

**Figure 8:**
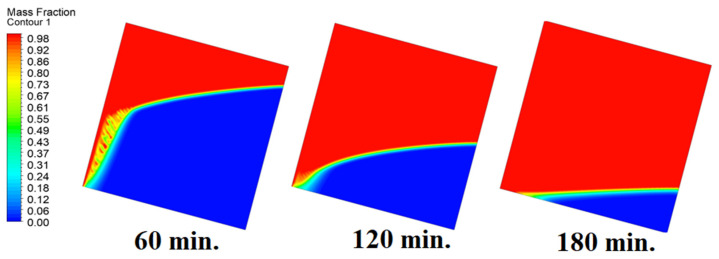
Progression of the melting process predicted with 15°.

**Figure 9:**
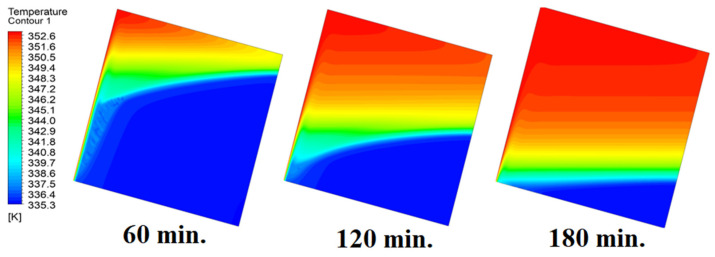
Temperature distributions against operational time with 15°.

**Figure 10:**
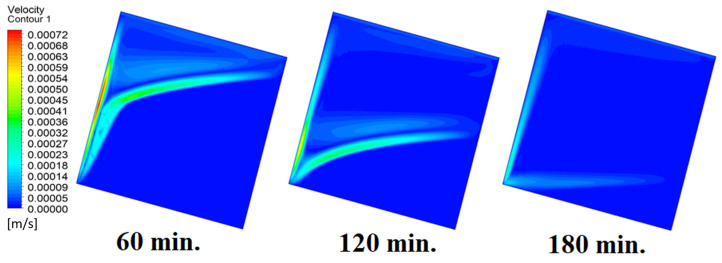
Velocity distributions against operational time with 15°.

In this case, the cell is studied at an angle of inclination of 15°. Figure 8 shows that the melting process begins with the influence of the conductive load that is along the wall, and then the melting process relies on the natural load, which is evident as the PCM moves away from the wall. Indeed, the melting process becomes slower as we move away from the wall because heat transfer depends on natural convection. More importantly, the angle of 15° has an influence on the melting process due to the effect of gravity on the materials. This specifically shows a greater effect of the heat source if compared to the normal position with zero angle of inclination. Figure 9 represents the temperature distribution against the operational time which can identify the heat transfer to the PCM with an angle of inclination of 15°. Here, it shows a faster heat transfer compared to zero angle of inclination because it depends on the conduction load, however, it slows down as we move away from the wall because it relies on the natural load. Figure 10 represents the movement of molten PCM, which is initially close to the wall and then moves to the stage of material change.

### 4.3. Case Three (Angle = 30°)

**Figure 11:**
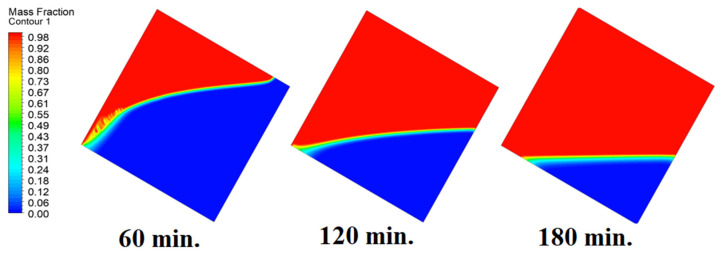
Progression of the melting process predicted with 30°.

**Figure 12:**
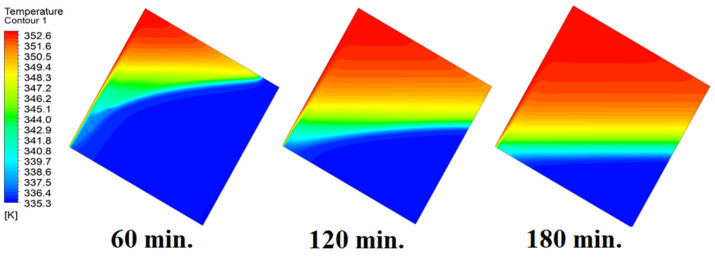
Temperature distributions against operational time with 30°.

**Figure 13:**
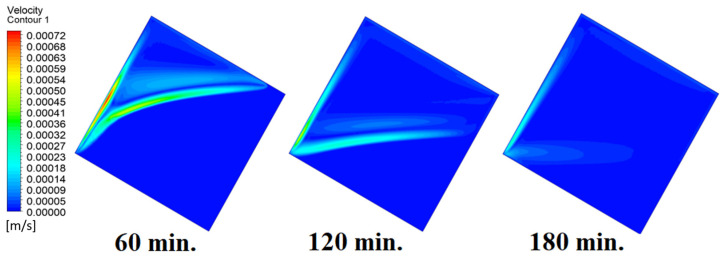
Velocity distributions against operational time with 30°.

In this case, the cell is studied at an angle of inclination of 30°. The same concept of having the effect of the conductive load on the melting process along the wall is noted in Figure 11. However, it is followed by the consequence of natural convection on the melting process as the PCM moves away from the wall and the melting process becomes slower. The increase of the angle of inclination from 15° to 30° has elucidated a progressive impact on the melting process of the PCM due to the increase of gravity, which deduces further effect of the heat source compared to 15° and 0°. Also, this elucidates an upsurge in the time necessitated to complete the melting process as the angle rises to 30°. Figure 12 and Figure 13 present the temperature and velocity distributions in the PCM at different operational times. The same findings are verified as represented in Figure 8 and Figure 9.

### 4.4. Case Four (Angle = 45°)

**Figure 14:**
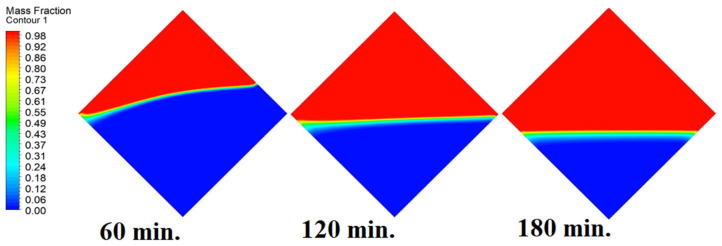
Progression of the melting process predicted with 45°.

**Figure 15:**
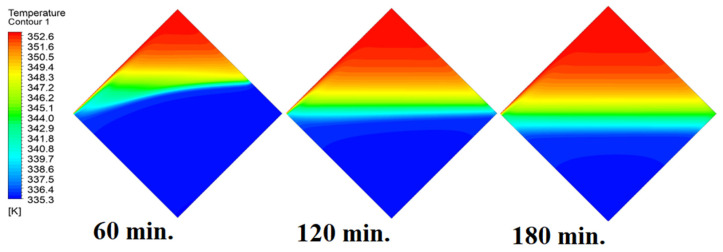
Temperature distributions against operational time with 45°.

**Figure 16:**
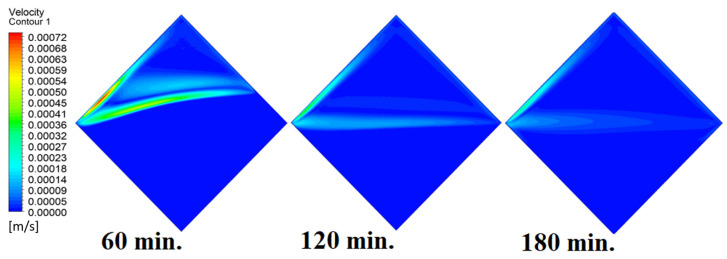
Velocity distributions against operational time with 45°.

In this case, the cell is studied at an angle of inclination of 45°. Figure 14, Figure 15 and Figure 16 introduce the evolution of the melting process, temperature distribution, and velocity distribution, respectively. Clearly, raising the angle of inclination to 45° aids in increasing the evolution of the melting process (Figure 14) compared to all the studied cases (0°, 15°, and 30°), which interprets the increase of the time necessitated to finalize the melting process. Also, Figure 15 and Figure 16 present the same behaviors that have been shown in the previous cases. Specifically, the conduction load causes the transfer of heat to PCM as it decreases from being away from the wall as it is affected by the natural load. Figure 16 also assures of having a uniform velocity distribution as a result of increasing the operational time.

**Figure 17:**
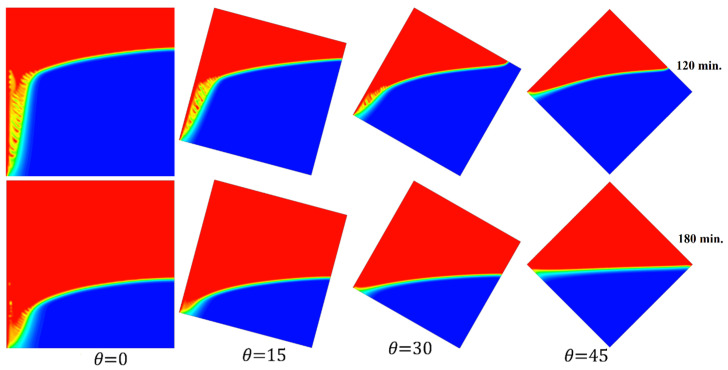
Comparison of the melting process of different angles of inclination.

Figure 17 elucidates the progression of the melting process for the tested cases of 0°, 15°, 30°, and 45° at 120 and 180 min in one go. This introduces the importance of having more time to finalize the melting process as the angle of inclination increases.

## 5. Evaluation of the Completion Time of the Melting Process for the Studied Cases

To perceive the influence of the angle of inclination of the cell on the melting process of PCM, it is essential to precisely compare the associated results of the considered cases of altered angles of 0°, 15°, 30°, and 45°. This in turn would interpret the influence of gravity on the melting process of PCM and the completion time of the melting process.

An examination of the PCM’s melting properties in square cells demonstrates a complex interaction of variables impacted by the inclination angle. The conduction channel is shorter at smaller angles (0°), which facilitates effective heat transfer and a speedier start to natural convection. An inclination-dependent attraction force is introduced by higher inclination angles, which affects the strength and patterns of natural convection. This has an impact on temperature gradients, magma migration, and the amount of time needed for a substantial material change.

During the presentation of variation of melting fraction against the operational time of different angles of inclination, there is a clear concavity of curve occurs using 45° of inclination angle between 100 to 200 min while the other inclination angles of 30° and 15° have such concavity with a lower intensity. Indeed, this suggests a noteworthy phenomenon in the melting process of PCM within square cells. One possible explanation for the concavity is a change in the major heat transmission pathway. At first, conduction is more common close to the heated wall, which causes the melting fraction to rise more sharply. Natural convection increases in importance over time, causing the concavity to gradually shift. A further explanation for the concavity could be the complicated nature of the fluid flow patterns inside the PCM at the designated inclination degree and time range. The concave shape may be caused by periodic changes in the melting fraction resulting from the interactions among ascending and descending currents.

**Figure 18:**
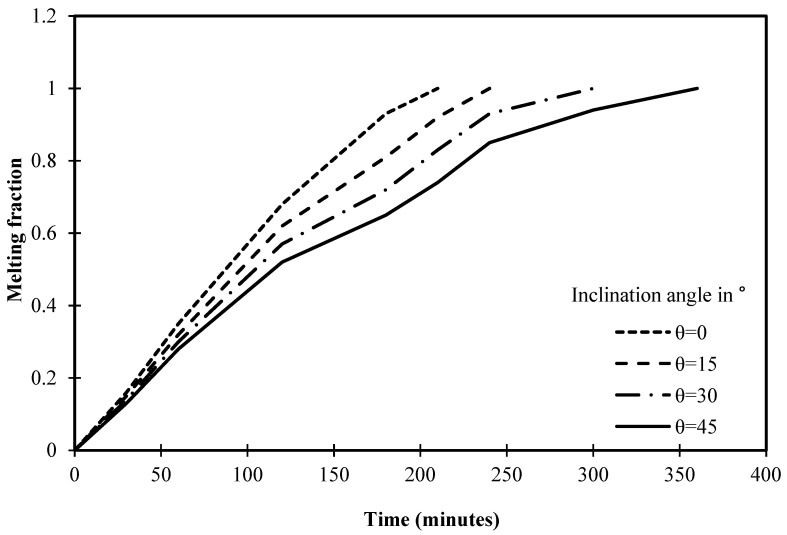
Variation of melting fraction against the operational time of different angles of inclination.

Figure 18 depicts that the melting process is accomplished in 210 min in the cell without an angle of inclination (0°). However, the melting process is completed in 240 min after utilizing the inclination angle of 15°. Thus, it is fair to admit that the melting process requires more time to complete the melting of PCM as the angle of inclination increases. In this regard, Figure 18 depicts that the melting process ends with angles of 30° and 45° at 300 and 360 min, respectively.

Statistically, it should be noted that the time required to achieve the melting process has increased by 15% when the inclination angle of the cell rises from 0° to 15°. However, the melting process of the cell has increased by 42% when the inclination angle of the cell is 30° if compared to 0°. In this regard, a significant increase in melting time has been deduced after utilizing 45° as it increases by 71% if compared to a cell that does not have an angle of inclination.

**Figure 19:**
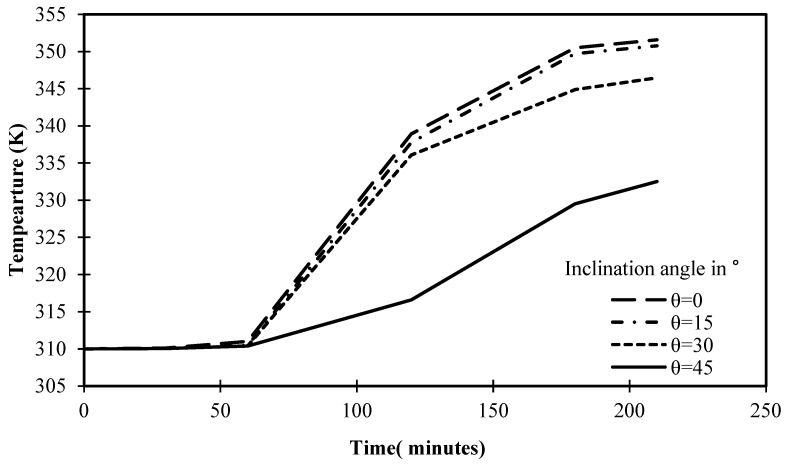
Variation of temperature against the operational time of different angles of inclination.

The progression of PCM temperature for various situations of angle of inclination is shown in Figure 19 for a set operational time of 210 min. It is obvious that the cells inclined at 0° and 15° had maximum temperatures of 351.5 K and 350.7 K, respectively, after 210 min. These consistent results can be easily contrasted with the 346.4 K value attained by the cell with a 30° angle of inclination in 210 min. With a 45° angle, the temperature of PCM is further reduced, reaching only 332.5 K. This is solid proof of how inclination angle affects heat transfer inside the cell and the melting process of PCM. Beyond this argument, Figure 19 introduces the closest temperature to equilibrium is 350 K, as the temperature begins to stabilize, turning solids into liquids, and reaching the maximum level of energy storage.

## 6. Conclusions

The present investigation employed ANSYS/FLUENT 16 software to perform a numerical analysis aimed at ascertaining the influence of the cell’s inclination angle on the melting of paraffin wax PCM (RT42) within a square cell. The results showed that the time necessitated to complete the melting process rose by 15% when the cell’s inclination angle climbed from 0° to 15° and by 42% and 71% when it proceeded from 0° to 30° and 45°, respectively. Furthermore, for 210 min of operation, the highest temperature of the PCM was 351.5 K with the use of a 0° angle of inclination compared to only 332.5 K with a 45° angle. In order to improve the system’s reactivity and suitability for application in real-world scenarios, engineers can use the current study to aid maximize heat transfer inside a PCM container while assuring rapid charging (melting) and discharging (solidification) operations.

One of the main limitations of the current study is the assumption of constant thermophysical parameters for paraffin wax PCM. This might not accurately reflect variances encountered in practice. Thus, it might be feasible to examine how non-uniform material qualities affect the PCM while taking into consideration potential differences found in real-world situations.

## Figures and Tables

**Table 1:** Thermo-physical properties of the Paraffin (RT42) [25].

Properties	RT42
Density, *ρ*	760 (kg/m^3^)
Specific heat capacity, *C_p_*	2 000 (J/kgK)
Thermal conductivity, *k*	0.2 (W/mK)
Dynamic viscosity, *μ*	0.02351 (kg/ms)
Thermal expansion rate, *α*	0.0005 (1/K)
Latent heat, *L*	165 000 (J/kg)
Melting temperature, *T_m_*	311.15–315.15 (K)

## Data Availability

Data are contained within the article.
